# Supplementary material for: A scoping review of the levels, implementation strategies, enablers, and barriers to cervical, breast, and colorectal cancer screening among migrant populations in selected English-speaking high-income countries
Source: PLoS One. 2025 Aug 14;20(8):e0329854. doi: 10.1371/journal.pone.0329854 (PMC12352849; doi:10.1371/journal.pone.0329854)
Supplement: S2 Table — (DOCX) [file pone.0329854.s002.docx]

S2 Table: Search strategy (databases, search domains and search terms)

| **Database** | **Search strings** |
| --- | --- |
| **Terms** | bowel OR colon OR colorectal OR rect* OR breast OR cervi* OR uterine  neoplas* OR cancer* OR malign* OR carcin* OR tumor* OR tumour* OR polyp* OR dysplasia OR adenocarcinoma  Screen* OR Detection OR Diagnosis OR detect*  “Asylum seeker*” OR CALD OR “culturally and linguistically diverse” OR “culturally and linguistically*” OR “culturally diverse” OR “diverse populations” OR “emigrants and immigrants” OR emigrant* OR “ethnic diversity” OR “ethnic minorities” OR ethnic OR “ethnically diverse” OR expatriate OR “foreign-born individuals” OR “minority and vulnerable populations” OR immigrant* OR migrant* OR newcomers OR “non-English-speaking-background” OR refugee* OR “transient migrants”  Uptake OR coverage OR Knowledge OR Attitudes OR Practice OR Behaviour OR behavior OR Accept* OR usage OR utilization OR utilisation OR use  Australia* OR Canada* OR “United Kingdom” OR “united states of America” OR “United States” OR “New Zealand” OR England OR Scotland OR Wales OR “Northern Ireland” |
| **PubMed** | ((((bowel[Title/Abstract] OR colon[Title/Abstract] OR colorectal[Title/Abstract] OR rect*[Title/Abstract] OR breast[Title/Abstract] OR cervi*[Title/Abstract] OR uterine[Title/Abstract]) AND (Screen*[Title/Abstract] OR Detection[Title/Abstract] OR Diagnosis[Title/Abstract] OR detect*[Title/Abstract])) AND ("Asylum seeker*"[Title/Abstract] OR CALD[Title/Abstract] OR "culturally and linguistically diverse"[Title/Abstract] OR "culturally and linguistically*"[Title/Abstract] OR "culturally diverse"[Title/Abstract] OR "diverse populations"[Title/Abstract] OR "emigrants and immigrants"[Title/Abstract] OR emigrant*[Title/Abstract] OR "ethnic diversity"[Title/Abstract] OR "ethnic minorities"[Title/Abstract] OR ethnic[Title/Abstract] OR "ethnically diverse"[Title/Abstract] OR expatriate[Title/Abstract] OR "foreign-born individuals"[Title/Abstract] OR "minority and vulnerable populations"[Title/Abstract] OR immigrant*[Title/Abstract] OR migrant*[Title/Abstract] OR newcomers[Title/Abstract] OR "non-English-speaking-background"[Title/Abstract] OR refugee*[Title/Abstract] OR "transient migrants"[Title/Abstract])) AND (Uptake[Title/Abstract] OR coverage[Title/Abstract] OR Knowledge[Title/Abstract] OR Attitudes[Title/Abstract] OR Practice[Title/Abstract] OR Behaviour[Title/Abstract] OR behavior[Title/Abstract] OR Accept*[Title/Abstract] OR usage[Title/Abstract] OR utilization[Title/Abstract] OR utilisation[Title/Abstract] OR use[Title/Abstract])) AND (Australia*[Title/Abstract] OR Canada*[Title/Abstract] OR "United Kingdom"[Title/Abstract] OR "united states of America"[Title/Abstract] OR “united States”[Title/Abstract] OR "New Zealand"[Title/Abstract] OR England[Title/Abstract] OR Scotland[Title/Abstract] OR Wales[Title/Abstract] OR "Northern Ireland"[Title/Abstract]) Filters: from 2015 - 2024 |
| **Scopus** | ( ABS ( bowel OR colon OR colorectal OR rect* OR breast OR cervi* OR uterine AND neoplas* OR cancer* OR malign* OR carcin* OR tumor* OR tumour* OR polyp* OR dysplasia OR adenocarcinoma ) AND ABS ( screen* OR detection OR diagnosis OR detect* ) AND ABS ( "Asylum seeker*" OR cald OR "culturally and linguistically diverse" OR "culturally and linguistically*" OR "culturally diverse" OR "diverse populations" OR "emigrants and immigrants" OR emigrant* OR "ethnic diversity" OR "ethnic minorities" OR ethnic OR "ethnically diverse" OR expatriate OR "foreign-born individuals" OR "minority and vulnerable populations" OR immigrant* OR migrant* OR newcomers OR "non-English-speaking-background" OR refugee* OR "transient migrants" ) AND ABS ( uptake OR coverage OR knowledge OR attitudes OR practice OR behaviour OR behavior OR accept* OR usage OR utilization OR utilisation OR use ) AND TITLE-ABS-KEY ( australia* OR canada* OR "United Kingdom" OR "united states of America" OR "United States" OR "New Zealand" OR england OR scotland OR wales OR "Northern Ireland" ) ) |
| **Embase** | (bowel:ab,ti OR colon:ab,ti OR colorectal:ab,ti OR rect*:ab,ti OR breast:ab,ti OR cervi*:ab,ti OR uterine:ab,ti) AND (neoplas*:ab,ti OR cancer*:ab,ti OR malign*:ab,ti OR carcin*:ab,ti OR tumor*:ab,ti OR tumour*:ab,ti OR polyp*:ab,ti OR dysplasia:ab,ti OR adenocarcinoma:ab,ti) AND (screen*:ab,ti OR detection:ab,ti OR diagnosis:ab,ti OR detect*:ab,ti) AND ((((('asylum seeker*':ab,ti OR cald:ab,ti OR culturally:ab,ti) AND 'linguistically diverse':ab,ti OR culturally:ab,ti) AND linguistically*:ab,ti OR 'culturally diverse':ab,ti OR 'diverse populations':ab,ti OR emigrants:ab,ti) AND immigrants:ab,ti OR emigrant*:ab,ti OR 'ethnic diversity':ab,ti OR 'ethnic minorities':ab,ti OR ethnic:ab,ti OR 'ethnically diverse':ab,ti OR expatriate:ab,ti OR 'foreign-born individuals':ab,ti OR minority:ab,ti) AND 'vulnerable populations':ab,ti OR immigrant*:ab,ti OR migrant*:ab,ti OR newcomers:ab,ti OR 'non-english-speaking-background':ab,ti OR refugee*:ab,ti OR 'transient migrants':ab,ti) AND (uptake:ab,ti OR coverage:ab,ti OR knowledge:ab,ti OR attitudes:ab,ti OR practice:ab,ti OR behaviour:ab,ti OR behavior:ab,ti OR accept*:ab,ti OR usage:ab,ti OR utilization:ab,ti OR utilisation:ab,ti OR use:ab,ti) AND (australia*:ab,ti OR canada*:ab,ti OR 'united kingdom':ab,ti OR 'united states of america':ab,ti OR 'united states':ab,ti OR 'new zealand':ab,ti OR england:ab,ti OR scotland:ab,ti OR wales:ab,ti OR 'northern ireland':ab,ti) |
| **Web of Science** | bowel OR colon OR colorectal OR rect* OR breast OR cervi* OR uterine neoplas* OR cancer* OR malign* OR carcin* OR tumor* OR tumour* OR polyp* OR dysplasia OR adenocarcinoma (Abstract) and Screen* OR Detection OR Diagnosis OR detect* (Abstract) and “Asylum seeker*” OR CALD OR “culturally and linguistically diverse” OR “culturally and linguistically*” OR “culturally diverse” OR “diverse populations” OR “emigrants and immigrants” OR emigrant* OR “ethnic diversity” OR “ethnic minorities” OR ethnic OR “ethnically diverse” OR expatriate OR “foreign-born individuals” OR “minority and vulnerable populations” OR immigrant* OR migrant* OR newcomers OR “non-English-speaking-background” OR refugee* OR “transient migrants” (Abstract) and Uptake OR coverage OR Knowledge OR Attitudes OR Practice OR Behaviour OR behavior OR Accept* OR usage OR utilization OR utilisation OR use (Abstract) and Australia* OR Canada* OR “United Kingdom” OR “united states of America” OR “United States” OR “New Zealand” OR England OR Scotland OR Wales OR “Northern Ireland” |
