# Supplementary material for: A scoping review of the levels, implementation strategies, enablers, and barriers to cervical, breast, and colorectal cancer screening among migrant populations in selected English-speaking high-income countries
Source: PLoS One. 2025 Aug 14;20(8):e0329854. doi: 10.1371/journal.pone.0329854 (PMC12352849; doi:10.1371/journal.pone.0329854)
Supplement: S3 Table — (DOCX) [file pone.0329854.s003.docx]

S3 Table: inclusion and exclusion criteria for selection of studies in the review

| **Domain** | **Inclusion criteria** | **Exclusion criteria** |
| --- | --- | --- |
| **Population** | Studies involving migrants eligible for bowel, cervical, or breast cancer screening in Australia, New Zealand, Canada, the USA, or the UK. | Studies focusing solely on populations outside included countries.  Studies involving populations with medical conditions precluding routine screening (e.g., individuals under active cancer treatment). |
| **Intervention/exposure** | Utilization of organized or opportunistic screening programs for bowel, cervical, or breast cancer.  Factors influencing participation in these screening programs (e.g., enablers, barriers). | Studies unrelated to bowel, cervical, or breast cancer screening services.  Studies addressing diagnostic procedures rather than preventive screening. |
| **Outcomes** | Utilization rates of bowel, cervical, and breast cancer screening services.  Enablers and barriers influencing screening uptake.  Best practices, interventions, or strategies to improve screening participation and service delivery. | Studies that do not report on utilization rates, enablers, barriers, or best practices.  Articles exclusively assessing clinical efficacy of screening methods without examining utilization or related factors. |
| **Study design** | Quantitative studies (e.g., cross-sectional, cohort, randomized controlled trials).  Qualitative studies (e.g., interviews, focus groups, case studies).  Mixed-methods studies combining quantitative and qualitative data. | Editorials, commentaries, opinion pieces, or conference abstracts lacking substantial data.  Studies with insufficient methodological quality or inadequate data to address the research questions. |
| **Geography** | Studies conducted in Australia, New Zealand, Canada, the USA, or the UK. | Studies conducted outside the specified countries. |
| **Publication year** | Studies published within the last 10 years to reflect practices and contexts. | Studies published more than 10 years ago unless deemed foundational or exceptionally relevant. |
| **Accessibility** | Full-text articles available. | Articles with only abstracts available or inaccessible full texts. |
