# Supplementary material for: A scoping review of the levels, implementation strategies, enablers, and barriers to cervical, breast, and colorectal cancer screening among migrant populations in selected English-speaking high-income countries
Source: PLoS One. 2025 Aug 14;20(8):e0329854. doi: 10.1371/journal.pone.0329854 (PMC12352849; doi:10.1371/journal.pone.0329854)
Supplement: S5 Table — (DOCX) [file pone.0329854.s005.docx]

S6 Table: Description of studies, design, population, country, migrants’ groups, and cancer types included in the studies.

| Characteristics | Categories | Frequency | % |
| --- | --- | --- | --- |
| Study design (n=80) | Quantitative | 40 | 50.0 |
|  | Qualitative | 33 | 41.3 |
|  | Mixed methods | 7 | 8.7 |
| Data collection (n=92) | Cross-sectional survey | 41 | 51.2 |
|  | Focus group discussions | 14 | 17.5 |
|  | Interviews | 25 | 31.3 |
| Study country (n=80) | USA | 38 | 47.5 |
|  | Australia | 20 | 25.0 |
|  | Canada | 15 | 18.7 |
|  | UK | 7 | 8.7 |
| Immigrants (n=80) | South Asian immigrants | 11 | 13.7 |
|  | East Asians | 8 | 10.0 |
|  | African immigrants | 16 | 20.0 |
|  | Middle eastern/Arab immigrants | 10 | 12.5 |
|  | Latino/Hispanic immigrants | 6 | 7.5 |
|  | Refugees- specific groups | 8 | 10.0 |
|  | European immigrants | 3 | 3.7 |
|  | CALD backgrounds | 5 | 6.2 |
|  | General immigrant populations, ethnic minorities | 13 | 16.2 |
| Location (n=80) | Clinics | 15 | 18.7 |
|  | Hospitals | 8 | 10.0 |
|  | Community Centers/Organizations | 12 | 15.0 |
|  | Religious Institutions | 6 | 7.5 |
|  | Urban Centers/Cities | 20 | 25.0 |
|  | Home-Based/Telephone/Online | 6 | 7.5 |
|  | Not Reported | 13 | 16.3 |
| Cancer types (n=80) | Cervical Cancer | 32 | 40.0 |
|  | Breast Cancer | 25 | 31.3 |
|  | Colorectal Cancer | 13 | 16.3 |
|  | Breast and Cervical Cancer | 6 | 7.5 |
|  | Breast and Colorectal Cancer | 2 | 2.5 |
|  | Cervical, Colorectal, and Breast Cancer | 2 | 2.5 |
